# The Human Gut Microbiome is Structured to Optimize Molecular Interaction Networks

**DOI:** 10.1016/j.csbj.2019.07.011

**Published:** 2019-07-29

**Authors:** Yiwei Ling, Yu Watanabe, Shujiro Okuda

**Affiliations:** Niigata University Graduate School of Medical and Dental Sciences, 1-757 Asahimachi-dori, Chuo-ku, Niigata 951-8510, Japan

**Keywords:** Microbiome, Molecular interaction networks, Human gut, Metagenomics, Interspecific interaction, KO, KEGG Orthology, IGC, Integrated reference catalog of the human gut microbiome

## Abstract

Microbiome studies estimate the functions of bacterial flora in situ on the basis of species composition and gene function; however, estimation of interspecies interaction networks is challenging. This study aimed to develop a method to predict the interaction networks among bacterial species from human gut metagenome data using bioinformatics methods. Our proposed method revealed that adjacent gene pairs involved in bacterial interspecies interactions are localized at boundary regions and encode membrane proteins mediating interactions between the intracellular and extracellular environments, e.g., transporters and channel proteins, and those mediating interactions between metabolic pathways. Actual human gut metagenome data displayed numerous such highly reliable interspecies interaction gene pairs in comparison with random simulated metagenome data sets, suggesting that the species composition of the actual microbiome facilitated more robust interspecific interactions. The present results indicate that molecular interaction networks in human gut flora are organized by a combination of interaction networks common to all individuals and group-specific interaction networks.

## Graphical Abstract

**Figure:**
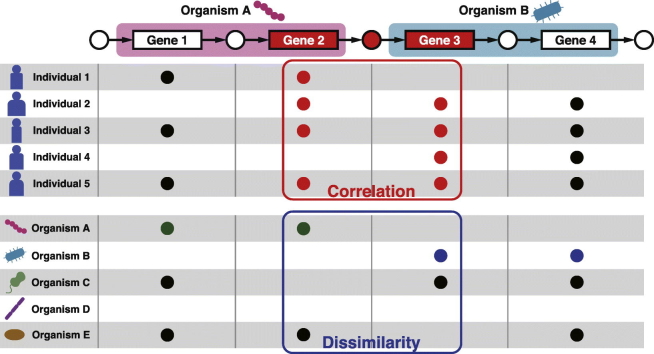

## 1 Introduction

Over a decade has passed since the culmination of large-scale microbiome studies [[1], [2], [3], [4], [5], [6], [7]]. Meanwhile, studies have described the organization of the microbiome and its functions in situ [2,[8], [9], [10]]. Microbiome species compositions reportedly vary among individuals; however, distributions of metabolic functions remain similar [4,7]. In other words, molecular interactions may mediate common functions in the human microbiome even for combinations of different species. Databases of biological pathways and networks are typically used to obtain information regarding such molecular interactions among bacteria. KEGG is one of most popular databases, presenting large-scale data regarding molecular interactions [11], and is widely used as a promising pathway database to observe and predict bacterial interactions. Furthermore, the STRING database contains data regarding molecular interactions from various sources [12], providing information regarding various intermolecular interaction networks including not only well-established biological knowledge and molecular interactions from experimental data but also correlative information inferred through text mining. Therefore, the STRING database is also frequently used as a reference database for interaction networks in microbiomes and for other “omics” studies. Other databases [13,14] are also used and a wide variety of models [[15], [16], [17]] are discussed to predict molecular interactions; nonetheless, it is difficult to predict genes and molecules involved in bacterial interactions in specific environments. Because most information in these databases is derived from experimental evidence of metabolic activity in specific species, functional data remain unclear owing to the heterogenous bacterial composition of microbiomes. In addition, this is one of the reasons for the dearth of evidence regarding specific metabolite intermediates for interspecific molecular interactions among microbiome bacterial species in environments unsuitable for the growth of certain species. Therefore, it is difficult to determine which molecular interaction in existing pathway networks should be focused on when considering molecular interactions among environmental bacteria.

In this study, we propose a new method to infer interaction networks among bacteria from microbiome data. We considered two associations in human gut microbiome data sets: phylogenetic profiles and co-occurrence profiles. To distinguish between bacterial cellular metabolism and molecular interactions among bacteria, inverse correlations among phylogenetic profiles were considered boundary regions of interspecific molecular interactions, along with correlations of co-occurrence profiles because species interactions occur within the gut of the same individual.

## 2 Materials & Methods

### 2.1 Data for Genomes and Pathway Networks

#### 2.1.1 The KEGG Database

The genomic data used herein was downloaded from KEGG (May 21, 2014) [11]. The ortholog identifiers in each genome, determined using KEGG Orthology (KO), were considered ortholog relationships. Furthermore, we used KEGG pathway data to estimate molecular interaction networks. Pathway map data excluding overview maps were extracted from kgml files as data from KEGG pathway maps. We derived relationships between “ecrel,” which defines enzyme reactions, and “map link,” connecting different pathway maps from kgml files, and reconstructed the entire pathway network. The adjacent KO pairs in the obtained network were considered candidate pairs to analyze molecular interactions among bacteria.

#### 2.1.2 Human Gut Metagenome Data

The Integrated reference catalog of the human gut microbiome (IGC) [5] was considered for actual human gut metagenomic data. All data were subjected to the same analysis pipelines for gene prediction, abundance estimation, and functional annotation. We downloaded the human gut metagenomic data derived from 324 healthy and 219 diseased individuals. Because of the version of KEGG used, we re-annotated all genes using KAAS (KEGG Automatic Annotation Server) [18] in accordance with KO. Furthermore, we used GhostX [19], which has a high calculation speed, to determine homology in the process of KAAS. From the re-calculated results, the relative abundance of each KO was re-determined based on the originally calculated values in the IGC project. In addition, as the relative abundance of genus level KO, the average value of relative abundance of KO confirmed for each genus was determined.

### 2.2 Interspecific Interaction Score

Based on genus level abundance obtained from IGC data, phylogenetic profiles were generated in accordance with the presence or absence of genera for each KO. Inverse correlations between the phylogenetic profiles and the reciprocal of the correlation coefficient (dissimilarity) among KOs were defined. In addition, as a definition of the co-occurrence relationship within the IGC sample, the correlation coefficient among all KO pairs was determined. As the interaction score of each KO pair (18,914 pairs with the intermediate compounds), the product of the dissimilarity of phylogenetic profiles and the correlation coefficient of the co-occurrence profile was calculated.

### 2.3 Validation of Interspecific Interaction

We defined a KO pair of metabolic relationships from pathway maps included in the category of “Metabolism.” To define the boundary between metabolic and non-metabolic relationships, we used the following three pathway maps: non-metabolic pathways, of 02010; ABC transporters, 02020; two-component system and 02060, and phosphotransferase system (PTS). We extracted relationships among a protein, its uptake compound, and a protein that produces the compound. These relationships between metabolic–metabolic and metabolic–non-metabolic KO pairs (see Supplementary Fig. S1) were used to validate the interspecific interaction score.

Pathway modules in KEGG Module defines metabolic pathways linking initial substrates and the final product. The initial substrates and the final products (edge) and intermediate metabolites (non-edges) were extracted from each module. To validate the interspecific interaction score, we enumerated the edge or non-edge intermediate metabolites between the interaction KO pairs.

### 2.4 Simulation of Metagenome Data

We extracted the KEGG genome data with the same genera as those in the IGC data. These genomes were randomly combined 1000 times to construct a random genome set. The number of genomes was randomly determined; however, only those sets containing unique KOs ranging between the minimum (3548 KOs) and maximum (6095 KOs) number of KOs of the IGC sample were considered. This was performed 1000 times in the same manner as a random genome set.

### 2.5 Data Availability

The source code of our method is available at http://bioinfo.med.niigata-u.ac.jp/csbj2019/.

## 3 Results

### 3.1 Organization of Interspecific Interaction Networks

**Fig. 1:**
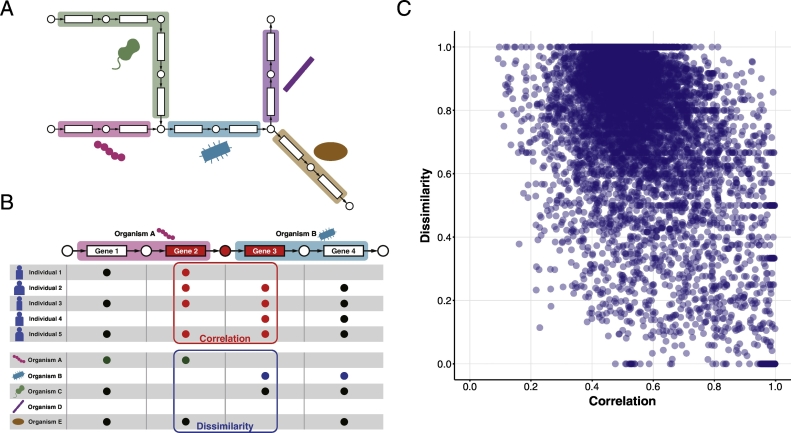
An overview of the reconstruction of interaction networks. (A) A schematic representation of the interspecific interaction networks of multiple organisms in different environments. (B) A model of our strategy to detect interspecific interaction boundaries based on the dissimilarity among phylogenetic profiles and correlation coefficients of co-occurrence profiles of adjacent gene pairs shared in two different species. (C) Correlation coefficient of co-occurrence profiles and dissimilarity of phylogenetic profiles among the gene pairs are plotted. The correlation coefficient of the distribution was −0.4771 and the R^2^ value was 0.2276.

Our objective was to reconstruct interaction networks among bacteria on the basis of data from each environment (Fig. 1A), for which phylogenetic and co-occurrence profiles (Fig. 1B) were used. If phylogenetic profiles of neighboring ortholog gene pairs on a pathway network show an inverse correlation, the ortholog gene pair can be considered a boundary of interactions between bacterial species possessing the genes. Conversely, the metabolic reaction catalyzed in this ortholog gene pair may represent a pathway not utilized in both bacterial species. Therefore, genes adjacent to those in the metabolic pathway showing inverse correlations among phylogenetic profiles were considered boundary candidates for interspecific interactions. Furthermore, correlations among co-occurrence profiles were also determined, because metabolism as an interspecific interaction can be realized only if these gene pairs are present in the human gut of the same individual. Finally, the product of the dissimilarity of the phylogenetic profile and the correlation coefficient of the co-occurrence profile was calculated as a score for identifying the interspecific interaction pairs (Fig. 1C and Supplementary Table S1).

### 3.2 Validation of Interspecific Interaction Scores

**Fig. 2:**
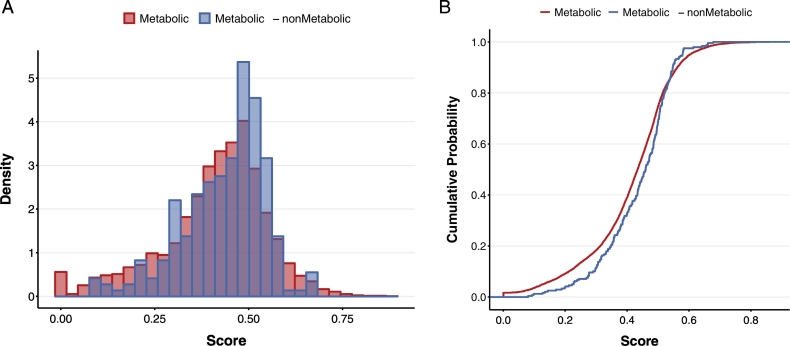
Distribution of interaction scores between metabolic and non-metabolic gene pairs. (A) The probability densities of the score distribution are visualized. (B) The cumulative probabilities of the score for metabolic gene pairs were significantly higher distributed (p-value = .00866, Kolmogorov-Smirnov test, one-sided).

In the obtained ortholog pair, two verifications were carried out to determine the threshold value of the score used to indicate an interaction. The first verification aimed to assess the interaction scores in boundary regions between metabolic pathways and non-metabolic pathways. Genes encoding cell-surface transporters and channel proteins possibly interact with other genes in other bacteria via some chemical compounds. Therefore, genes encoding transporters and channel proteins were considered the boundary between intracellular and extracellular metabolism. Genes in another bacterial cell linked via a compound produced from the genes on the boundary are regarded as non-metabolic genes. Thus, these gene pairs were defined as metabolic–non-metabolic gene pairs. A total of 239 such gene pairs were found. Conversely, pairs of enzyme-coding genes in other normal metabolic pathway maps were defined as metabolic pairs (a total of 8227 pairs), and differences in scores between these two ortholog pairs were observed. As shown in Fig. 2A and B, the metabolic–non-metabolic pairs had significantly higher interaction scores than the metabolic–metabolic pairs (p-value = .00866, Kolmogorov-Smirnov test, one-sided). These results suggest that ortholog gene pairs with the higher interaction scores were often localized at boundary regions of the interaction with extracellular regions.

### 3.3 Relationship between Interaction Scores and Pathway Modules

**Fig. 3:**
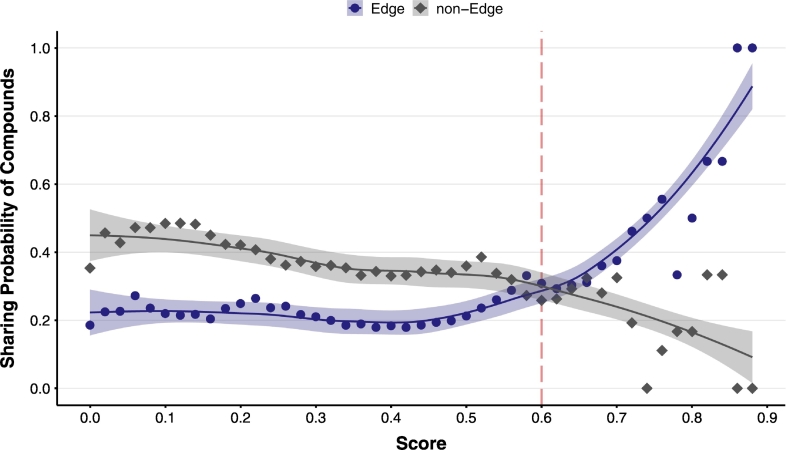
Relationship between the interaction scores and module linkage. Compounds at the start and end points (edge) and intermediate compounds (non-edges) were extracted from each module in the KEGG Module database. The number of interaction pairs with common compounds was determined, and the possibility of a common compound in each sore window was determined. If the score is >0.6, the edge compounds are significantly likely to be shared in the interaction gene pairs, which would possibly constitute greater module linkage.

We attempted to verify the score by using pathway modules. Therefore, it may be generally presumed that the units of this single module are complete in the metabolic pathways in the same species. Therefore, we distinguished the molecules at the beginning and end of the module and those within the module. We verified which scores of these two types of compounds exist between the interaction pairs. As shown in Fig. 3, when the interaction score for ortholog pairs is high (score > 0.6), the pairs significantly contributed to the metabolic pathway via compounds at both ends of the modules (p-value = 7.957e-05, Mann-Whitney test). Ortholog pairs with a high score (> 0.6) probably play a role in connecting modules with one another.

### 3.4 Comparison of IGC Data and Simulated Metagenome Data

**Fig. 4:**
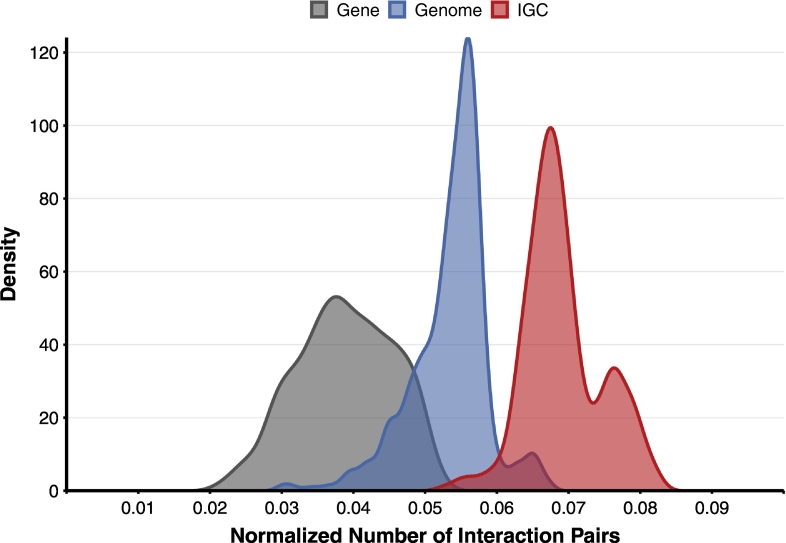
Distribution of the number of interaction pairs. Distribution of the number of the interaction gene pairs are plotted from actual human gut data (IGC), random genome sets (Genome), and random genes (Gene). The number of the interaction pairs was normalized by the number of total unique ortholog (KO) genes in each group. The IGC group has significantly large number of interactions than the other two sets (p-value <2.2e-16, ANOVA for the three groups; p-value <2.2e-16, Mann-Whitney test for IGC and both random sets).

Based on the aforementioned results, we decided to use the ortholog pairs with a score of 0.6 or higher as gene pairs highly likely to interspecifically interact, for subsequent analysis. To observe the interaction network defined herein, we compared the actual human gut bacterial metagenomic data with the simulated random metagenomic data. The metagenomic data of healthy individuals in the IGC database was considered the actual metagenomic data. Among the genomes annotated in KEGG, a set randomly combined with those restricted to organisms appearing in the IGC data was considered a random genome set. Furthermore, a random gene set was derived by randomly extracting genes from a unique gene set appearing in the random genome set. Fig. 4 shows a comparison between these two types of simulated metagenomic data with the actual metagenomic data from the human gut. Consequently, the actual metagenomic data from the human gut displayed the most interspecific interaction pairs. Thereafter, the random gene set displayed the lowest frequency with the interaction pairs. These results indicate that many bacterial species interact significantly with one another in the actual human gut environment (p-value <2.2e-16, ANOVA for the three groups; p-value <2.2e-16, Mann-Whitney test for IGC and both random sets).

### 3.5 Clustering for IGC Data

**Fig. 5:**
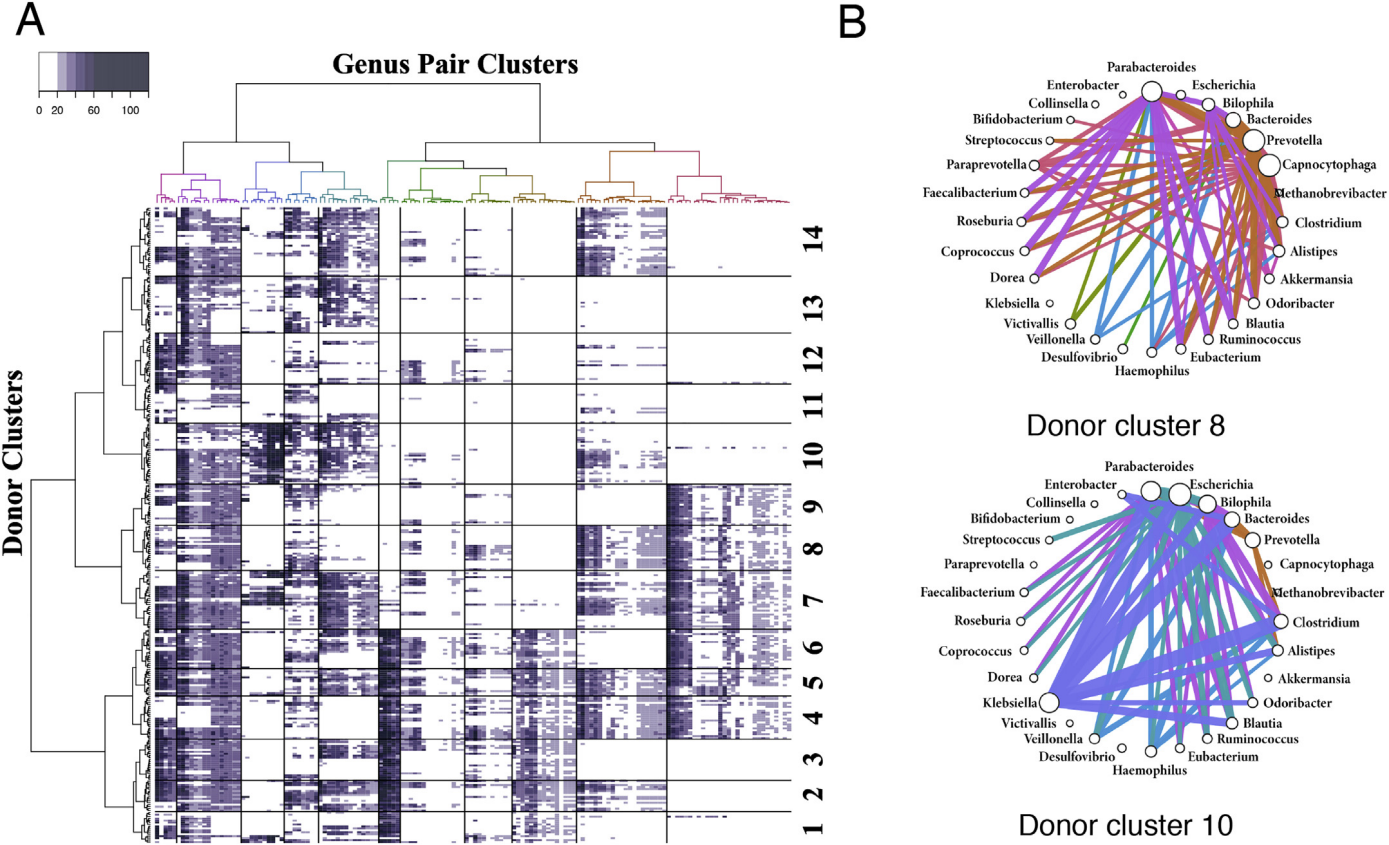
Interaction networks in the human gut microbiome data. (A) Donor groups differently characterized by common genus pairs were subjected to hierarchical clustering. Each genus cluster is colored. Genus pairs common to almost all donors were excluded from the clustering shown in Fig. S1. (B) Each donor cluster displayed specific interaction networks at the genus level. Donor clusters 8 and 10 are shown as prototype donor clusters in Fig. 5A. The colored edge represents a genus pair cluster (colored as in Fig. 5A), and the width represents the average score of the genus pair (an edge with <15 is not displayed). The size of a circle of the genus represents the total scores of the related genus pairs.

To assess the organization of interaction networks in the human gut metagenome of an individual, a hierarchical clustering with the IGC data was performed. Prior to the clustering, we assigned orthologs to each genus level and converted the ortholog pairs to genus pairs, because the number of interaction pairs is large, and we performed hierarchical clustering for the genus pairs (Supplementary Fig. S2). Based on these clustering results, the interacting genus pairs were divided into those common to almost all individuals and the genus pairs only present in specific groups of some individuals. In addition, re-clustering was performed using data based on pairs appearing only in specific individual donor groups, except for those common to almost all individuals (Fig. 5A). Consequently, the donor cluster was divided into 14 groups. Because the interaction networks of the genus pairs are expected to differ for each cluster, the linkage of each genus pair is visualized as a network diagram (Fig. 5B and Supplementary Fig. S3). Consequently, there was a difference in the linkage of the interaction network in the human gut environment among individuals.

### 3.6 Comparison between Healthy and Disease Samples

To compare interaction scores between healthy and disease samples, we also calculated the score using metagenomic data in disease samples labeled in the IGC data (Supplementary Table S2). Subsequently, 8352 KO pairs present in both healthy and disease groups were extracted. To search for specific KO pairs with differences between healthy and disease groups, two standard deviations from the mean of score differences between them were used as a threshold. We extracted KO pairs with an interaction score >0.6 to define KO pairs specific to healthy and disease samples (Supplementary Fig. S4). As a result, 76 and 107 KO pairs were found from healthy and disease samples, respectively (Supplementary Table S3).

## 4 Discussion

This study evaluated interspecific interaction networks of the human gut microbiome based on the scoring method developed herein with bacterial interaction pairs, displaying robust bacterial species interactions in the actual human gut environment. The present results suggest that the actual human gut environment probably retains significantly more bacterial interactions than simple combinations of random genomes, and the human gut environment is at least a highly structured community, being responsible for environmental metabolic function. A high possibility of microbiome species not organized randomly but rather structurally organized has been speculated on the basis of interspecific interactions, thus yielding robust environmental perturbations. This study only utilized intestinal microbiomes to validate this speculation; however, this method may be used to evaluate the robustness in other environments such as the ocean and the soil. Comparison of the robustness of various environments should is important, considering the ecology of environmental microorganisms.

Our method uses the correlation of co-occurrence relationships and the inverse correlation of phylogenetic profiles to infer interspecific interactions for adjacent gene pairs on the pathway. In past reports, several studies focused mainly on the correlation of co-occurrence relationships (for example, SparCC [20]), but the concept of applying inverse correlation of phylogenetic profiles in addition to the correlation is an advantage of our method. Correlation-based methods are conceptually similar in terms of using co-occurrence correlation. However, by inferring the boundary of bacterial interactions using inverse correlation of phylogenetic profile, our method more specifically predicts the interspecific interaction as compared to other correlation-based methods.

This study focused on interactions among bacteria; however, bacteria-host interactions are also critical. Bacterial metabolites are reportedly absorbed by the human intestinal tract and utilized [[21], [22], [23]], and are involved in disease pathogenesis and progression [8,9,24,25]. In particular, the impact on the host immune system is well known [9,25]. Therefore, future studies are required to further establish analytical methods focusing on bacteria-host interactions. From the intermediate metabolites of interaction pairs with the scoring method proposed herein, high scores are mostly important for host metabolism such as amino acids and folic acid in the case of healthy samples. When we applied our approach to the differences between healthy and disease samples, we obtained interaction gene pairs and their intermediate compounds specific for each sample. For example, the KO pair via nitrite showed very high interaction scores in healthy samples but lower in disease samples. Nitrate plays an important role in the nitrogen cycle, and catalysis to ammonia via nitrite is performed by several enterobacteria such as *Escherichia coli* and lactic acid bacteria [26]. Furthermore, surprisingly, benzoate also showed a higher score as a healthy sample-related chemical compound. This compound seemed to be detected at higher levels in healthy feces (168.49 nmol/g wet in average) than in disease feces (not quantified) as described in the Human Metabolome Database (HMDB) [27]. Although chemical compounds such as hippurate synthesized from benzoate would be generally considered toxic to human body [28,29], a healthy gut microbiome community may use interspecific metabolic interactions to degrade it. To characterize the many interspecific interaction networks identified, future studies are required to focus on metabolic networks between bacteria and also their hosts on the basis of such intermediates.

Finally, although we validated our method using randomized data, we could still not perform any validation using real interaction data obtained from experiments such as transcriptome and metabolome analysis and single cell-based assays. Recently, real bacterial interspecies interaction has been reported based on such omics approach and co-culture experiments [30,31]. Verification using such real interaction data should be performed in a future study.

## 5 Conclusions

This study shows interactions among gene pairs, which organize bacterial interaction networks in a complex human gut environment. Comparison with random metagenomic simulation data suggests that the actual intestinal metagenome would comprise robust interspecific bacterial interaction networks. Furthermore, the intestinal environment of individuals is more likely to be optimized with a specific interaction network along with a common interaction network module.

## Declaration of Competing Interest

S.O. received consultant fees from Denka Co., Ltd. L.Y., and Y.W. have no conflict of interest.

## Appendix A Supplementary data

Supplementary material 1 Image 1

Supplementary material 2 Image 2
