# Supplementary material for: The Human Gut Microbiome is Structured to Optimize Molecular Interaction Networks
Source: Comput Struct Biotechnol J. 2019 Jul 29;17:1040–6. doi: 10.1016/j.csbj.2019.07.011 (PMC6700418; doi:10.1016/j.csbj.2019.07.011)
Supplement: Supplementary file 1 — Supplementary material 1 [file mmc1.pdf]

Supplementary materials

Human gut microbiome is structured to optimize molecular interaction networks

Yiwei Ling[a,1], Yu Watanabe[a,1], and Shujiro Okuda[a]

a) Niigata University Graduate School of Medical and Dental Sciences, 1-757 Asahimachi-dori, Chuo-ku, Niigata 951-8510, Japan

1) These authors contributed equally to this work

Corresponding author:
Shujiro Okuda
Niigata University Graduate School of Medical and Dental Sciences
1-757 Asahimachi-dori, Chuo-ku, Niigata 951-8510, Japan
E-mail: okd@med.niigata-u.ac.jp
Tel: +81-25-227-0390
Fax: +81-25-227-0393

Supplementary Figure S1

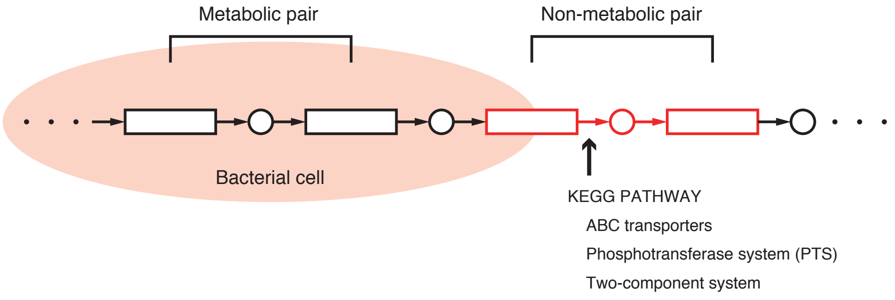

Genes encoding cell-surface transporters and channel proteins possibly interact with other genes in another bacteria via some chemical compounds. Therefore, these genes such as transporters and channel proteins were considered the boundary between intracellular and extracellular metabolism. Genes in another bacterial cell linked via a chemical compound produced from the genes on the boundary is regarded as non-metabolic genes. Thus, these gene pairs were defined as metabolic–non-metabolic gene pairs. Conversely, pairs of enzyme-coding genes in other normal metabolic pathway maps were defined as metabolic pairs.

Supplementary Figure S2

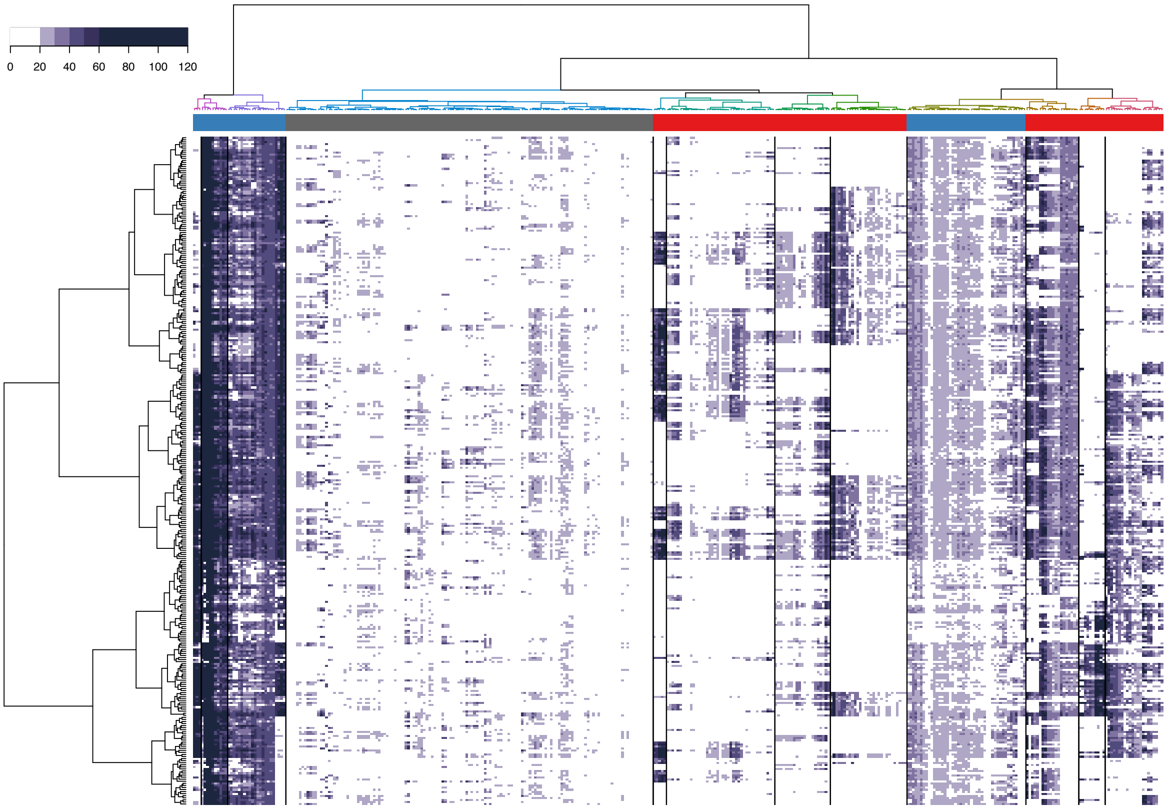

Hierarchical clustering of genus pairs. Note that genus pairs were excluded if the score with less than 10 and are common among more than 80% individuals. The commonly shared genus pair clusters (blue bar) and a randomly shared noise cluster (gray bar) could be distinguished owing to their uniform distribution. The genus pairs in these clusters were excluded from subsequent hierarchical clustering indicated in Fig. 5A.

Supplementary Figure S3

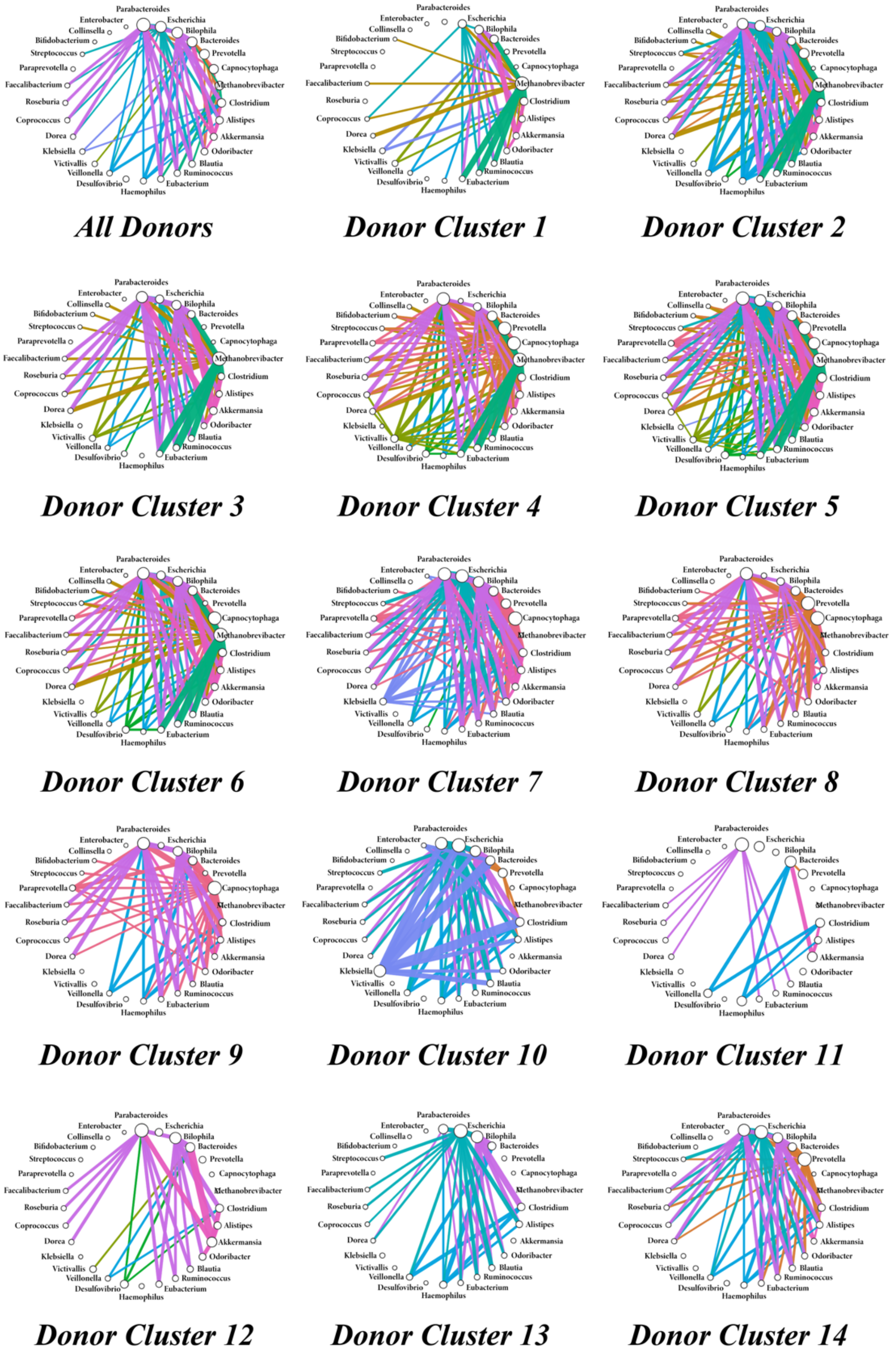

Interaction networks of each donor cluster. The score of genus pairs in each donor cluster are shown as linkages among genus networks in accordance with clustering indicated in Fig. 5A. Donor clusters shown here correspond to those in Fig. 5A. The color of edge is a genus pair cluster, and the width is the average score of the genus pair (an edge with less than 15 is not displayed). The size of a circle of genera represents the total scores of the related genus pairs.

Supplementary Figure S4

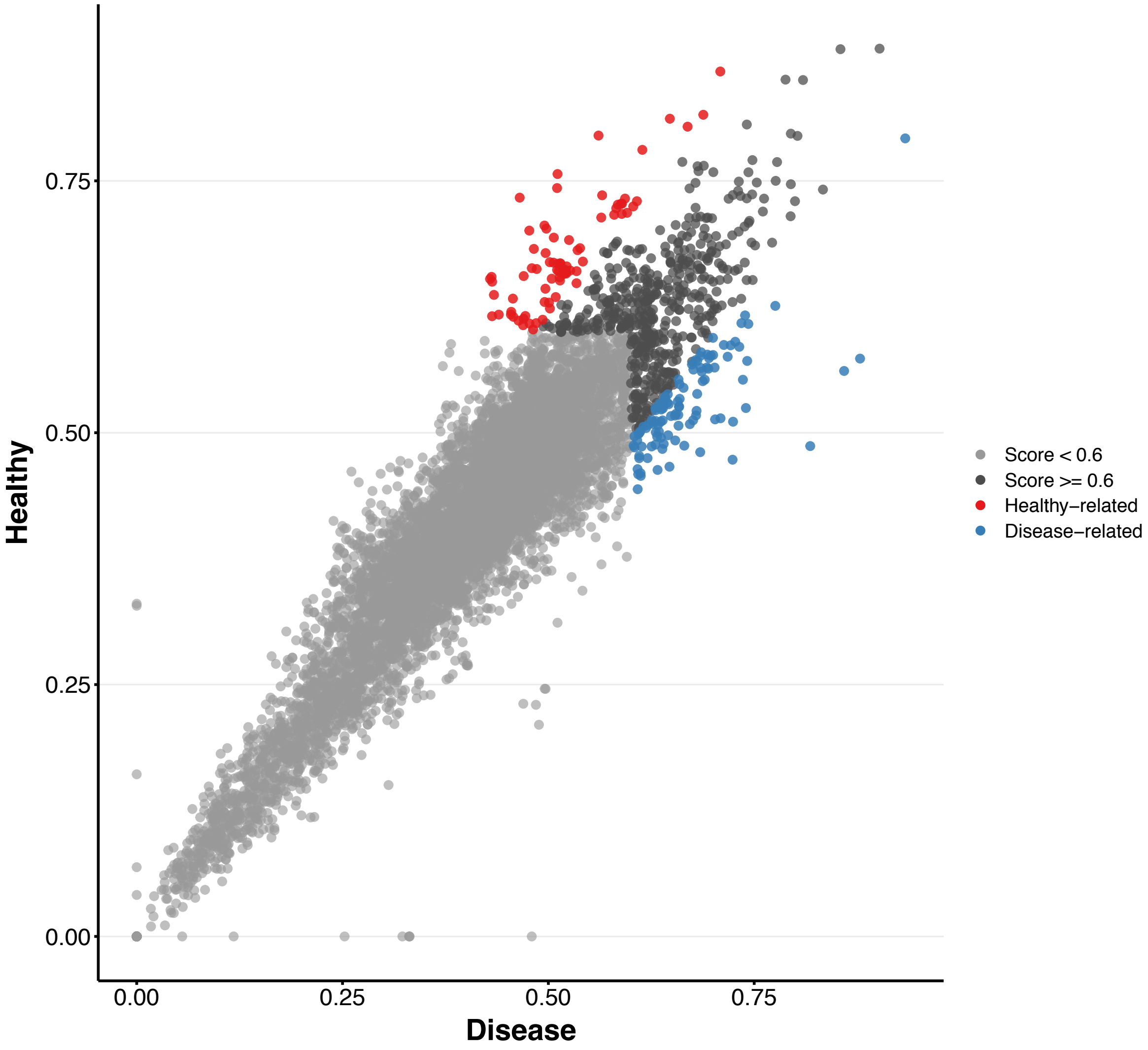

The interaction score are plotted for 8352 KO pairs both involved in healthy and disease groups. KO Pairs with more than two standard deviations from the mean of score differences between disease and healthy groups were colored blue and red, respectively. The other KO pairs with interaction score greater than equal to 0.6 is colored dark gray and those with less

than 0.6 is light gray.
